# Capillary electrophoresis-mass spectrometry for the direct analysis of glyphosate: method development and application to beer beverages and environmental studies

**DOI:** 10.1007/s00216-020-02751-0

**Published:** 2020-06-10

**Authors:** Benedikt Wimmer, Martin Pattky, Leyla Gulu Zada, Martin Meixner, Stefan B. Haderlein, Hans-Peter Zimmermann, Carolin Huhn

**Affiliations:** 1 Institute for Physical and Theoretical Chemistry, Eberhard Karls Universität Tübingen, Auf der Morgenstelle 18, 72076 Tübingen, Germany; 2 Center for Applied Geosciences, Environmental Mineralogy and Chemistry, Eberhard Karls Universität Tübingen, Hölderlinstr. 12, 72074 Tübingen, Germany; 3 Agilent Technologies, Hewlett-Packard-Str. 8, 76337 Waldbronn, Germany

**Keywords:** Matrix tolerance, Transient isotachophoresis, Food, Preconcentration

## Abstract

In this study, we developed and validated a CE-TOF-MS method for the quantification of glyphosate (*N*-(phosphonomethyl)glycine) and its major degradation product aminomethylphosphonic acid (AMPA) in different samples including beer, media from toxicological analysis with *Daphnia magna*, and sorption experiments. Using a background electrolyte (BGE) of very low pH, where glyphosate is still negatively charged but many matrix components become neutral or protonated, a very high separation selectivity was reached. The presence of inorganic salts in the sample was advantageous with regard to preconcentration via transient isotachophoresis. The advantages of our new method are the following: no derivatization is needed, high separation selectivity and thus matrix tolerance, speed of analysis, limits of detection suitable for many applications in food and environmental science, negligible disturbance by metal chelation. LODs for glyphosate were < 5 μg/L for both aqueous and beer samples, the linear range in aqueous samples was 5–3000 μg/L, for beer samples 10–3000 μg/L. For AMPA, LODs were 3.3 and 30.6 μg/L, and the linear range 10–3000 μg/L and 50–3000 μg/L, for aqueous and beer samples, respectively. Recoveries in beer samples for glyphosate were 94.3–110.7% and for AMPA 80.2–100.4%. We analyzed 12 German and 2 Danish beer samples. Quantification of glyphosate and AMPA was possible using isotopically labeled standards without enrichment, purification, or dilution, only degassing and filtration were required for sample preparation. Finally, we demonstrate the applicability of the method for other strong acids, relevant in food and environmental sciences such as *N*-acetyl glyphosate, *N*-acetyl AMPA (present in some glyphosate resistant crop), trifluoroacetic acid, 2-methyl-4-chlorophenoxyacetic acid, glufosinate and its degradation product 3-(methylphosphinico)propionic acid, oxamic acid, and others.

**Electronic supplementary material:**

The online version of this article (10.1007/s00216-020-02751-0) contains supplementary material, which is available to authorized users.

## Introduction

Glyphosate (GLP) is a widely used broadband herbicide with an annual production of more than one million tons [1]. Various applications exist including pre-emergence use up to desiccation before harvest, as discussed, e.g., by Schmitz and Garvert [2] for Germany. GLP blocks the shikimate pathway which is vital to plants for the synthesis of aromatic amino acids. Despite the fact that GLP is the most widely applied herbicide worldwide [3, 4], a thorough understanding of its fate in the environment including mobility, bioavailability, and microbial degradation is still lacking. In the environment, GLP can sorb to soil minerals which may limit bioavailability and thus prevent quantitative mineralization. GLP readily forms chelate complexes with di- and trivalent metal ions, resulting in a large variety of possible GLP species at environmental pH, altering its environmental behavior, not yet well understood [5]. Maximum legal contaminant limits in water largely differ between EU with 0.1 μg/L and the USA with 700 μg/L [6]. An overview on findings of GLP in water samples was summarized in the reviews by Vereecken [7] and Saunders and Pezeshki [6], and for AMPA by Grandcoin et al. [8]. Human dietary exposure to GLP was discussed by Stephenson and Harris [9], who also presented a large overview on GLP findings in foodstuff.

One major reason for the relatively limited knowledge on the environmental fate and impact of GLP and its main metabolite aminomethylphosphonic acid (AMPA) and difficulties of their quantification in foodstuff stems from their physicochemical properties, making their sensitive and precise analysis difficult (e.g., [10, 11]). GLP contains two acidic (phosphonic acid and carboxylic acid) and one basic (secondary amine) moieties, which make the molecule highly polar and doubly charged at the pH range of approximately 6–8 relevant for most surface water samples (p*K*_a_ values given in the literature 2.0/-/0.88, 2.6/2.32/2.22, 5.6/5.86/5.87, and 10.6/10.86/10.89; from [12–14]). Similarly, AMPA has a phosphonic acid and a primary amine as functional groups. Thus, its effective charge number at pH 6–8 values ranges from − 0.5 to − 1 (p*K*_a_ values [15] 1.85/2.35, 5.35/5.9, and 10.0/10.8). GLP’s high polarity and high charge at intermediate pH, complex speciation regarding pH and complexation/chelation with multivalent metal cations, ability to adsorb on surfaces (mineral oxides but also fused silica), and the lack of suitable chromophores pose significant challenges to most if not all analytical techniques. Koskinen et al. [16] presented an overview of analytical techniques and their application to different sample types (water/aqueous fluids, plant material, and soil). Sensor methods and electromigrative separation techniques were summarized by Gauglitz et al. [11]. Currently, gas chromatography (GC) with MS, electron capture, nitrogen phosphorous or flame ionization detection, and liquid chromatography (LC) with fluorescence detection or coupled to mass spectrometry are commonly used for sensitive GLP analysis [17, 18]. However, the polarity of GLP prevents its direct analysis by GC or reversed phase LC. These methods require a laborious and expensive derivatization procedure to provide the derivative with sufficient volatility, thermal stability, and sufficient retention, and to allow sensitive detection [19]. For RPLC, mostly 9-fluorenylmethoxycarbonyl chloride (FMOC) is used, but various other label reagents may be used [19]. Derivatization may be impaired by metal ions present in the sample matrix. This effect can be reduced by adding ethylenediaminetetraacetic acid (EDTA) to evoke competitive metal cation chelation, as envisaged in the ISO method 16308 [20]. These complexes impair derivatization yield, most likely due to changing the basicity of the glycyl group or having it involved in the ligand sphere rendering it less active for electrophilic attack by the FMOC reagent [21]. However, also contradicting results were reported [22]. Even after derivatization, metal cations are problematic as the FMOC-GLP derivative was shown to form very stable complexes with divalent metal cations, which are separated in RPLC [22]. The selectivity of the derivatization process is limited as all primary and secondary amines present, e.g., in food samples may be co-derivatized. Side reactions are possible and may lead to isobaric interferences [23, 24]. Accordingly, sample preparation techniques for matrix removal are employed, e.g., SPE with various materials including ion exchange resins [21, 25] or liquid-liquid extraction [26]. Direct GLP analysis without derivatization using LC was achieved using mixed mode columns, ion exchange chromatography, or hydrophilic liquid interaction chromatography [10]. Another possibility for direct GLP analysis taking advantage of its high charge is ion chromatography. IC-MS/MS was applied to quantify GLP in lettuce, oranges, and wheat flour extracts [27] within 10 min separation time. The instrumental setup is complex with a metal-free ion chromatograph, eluent generator (to produce KOH as eluent), and an electrochemically regenerated suppressor to replace cations from eluent and sample by H^+^ prior to the MS. Strong matrix effects were observed. LOD was 2.5 μg/L in the extract from 10 g sample. Also two-dimensional ion chromatography coupled to mass spectrometry was used requiring a further pump and suppressor [28]. The instrumentation required a generation chamber to produce KOH as eluent, two IC separation columns, a concentrator, and two suppressors prior to the MS instrument. With this setup, LODs down to 0.05 μg/L were reached, however, with analysis times of 30 min. Matrix effects were briefly discussed using a model sample made of salt solutions.

In contrast to chromatographic techniques, electromigrative separation techniques take advantage from the fact that GLP is negatively charged over a broad pH range [11]. Various direct methods without derivatization were published using indirect UV [29–32], fluorescence [33], capacitively coupled contactless conductivity detection [31, 34–37], inductively coupled plasma-MS [38], or ESI-MS [39–43], though applications with the latter coupling are still scarce. It is interesting to note that the full pH range has been used for the background electrolyte (BGE) starting from 2.45 [40] to 10 [42]. However, as also shown in this study, adsorption on the capillary wall might be problematic. Long rinsing protocols with strong acids or bases (e.g., [30, 32, 44–49]) and permanent [39, 41, 43, 50] or dynamic coatings [29–32, 34, 35, 51–53] have been used to improve precision and resolution. As for chromatographic techniques, derivatization of GLP and AMPA was conducted using various amine-based labeling reagents, e.g., fluorescein isothiocyanate (FITC) [45, 46, 52, 54, 55], FMOC [49, 56, 57], and others [44, 55, 58]. The limited resolution was overcome using cyclodextrins [46] or detergents in micellar electrokinetic chromatography (MEKC) [44, 45, 53, 54, 57–59]. UV detection at low wavelengths [49, 53, 56, 57, 60] or LIF detection, mostly at 488 nm [44–46, 52, 54, 55, 58, 61], was often used for detection.

Literature reveals a high matrix tolerance of CE methods for GLP analysis. When combined with a selective detection method such as LIF or MS, impressive results were obtained for complex samples including environmental samples such as ground water and surface water [32, 33, 35, 37, 38, 44, 46, 49, 51, 55, 56, 60] or soil [41, 55, 61], but also food such as beverages [41, 42, 50], soy or wheat products [29, 39, 43, 57, 62], and vegetables or fruits [43, 52, 59]. Furthermore, herbicides and reaction mixtures from herbicide production [33, 40, 42, 51], marijuana seizures [53], and human serum [63] were analyzed. Most studies used spiked samples to demonstrate the matrix tolerance of the methods. Online preconcentration methods for GLP analysis using CE presented so far comprise acetonitrile stacking [52, 62], field-amplified sample stacking [29], large volume sample stacking [29, 47], and column-coupled isotachophoresis (both in capillaries [36] and on-chip [48]). Also, field-amplified electrokinetic injection was used [29, 34, 57], however, with low matrix tolerance regarding the salt matrix of many samples, including surface water [29, 34].

In this study, we developed a direct (derivatization-free) highly selective and rapid analytical method for quantification of GLP and AMPA using CE-QTOF-MS. The method proved to be applicable for a large variety of matrices including beer beverages and aqueous samples from environmental sorption studies. Online sample preconcentration is discussed as well as matrix effects. Additionally, we demonstrate the method’s potential to analyze further pollutants/contaminants relevant for food and environmental samples such as *N*-nitroso glyphosate (NNG), *N*-acetyl glyphosate (NAG), *N*-acetyl AMPA (NAA), glufosinate (GLU), 3-(methylphosphinico)propionic acid (3-MPPA), oxamic acid, 2-methyl-4-chlorophenoxyacetic acid (MCPA), difluoroacetic acid (DFA), and trifluoroacetic acid (TFA), phosphonic acid and iminodiacetic acid (IDA).

## Materials and methods

### Chemicals

Isopropanol (LC-MS grade), methanol (LC-MS grade), glyphosate (> 99.7%), glufosinate ammonium salt (> 98%), and lead(II) nitrate (> 98%) were purchased from Fluka (Steinheim, Germany). Formic acid (FA) (98–100%, LC-MS LiChropur), formic acid-d2, sodium hydroxide (30%, Suprapur), cadmium(II) chloride monohydrate (> 98%), nickel(II) chloride hexahydrate (> 98%), calcium chloride dihydrate (> 99.5%), and copper(II) chloride dihydrate (> 99%) were bought from Merck (Darmstadt, Germany). Hydrochloric acid (32%, analytical grade) was obtained from Fisher Scientific (Schwerte, Germany). Twenty-five percent aqueous ammonia solution (p.a. grade), aluminum chloride, iron(III) chloride hexahydrate, manganese(II) chloride tetrahydrate, magnesium chloride hexahydrate (99.6%), zinc acetate dihydrate (> 99%), aminomethylphosphonic acid (> 99%), 2-methyl-4-chlorophenoxyacetic acid (99.2%), oxamic acid (> 98%), difluoroacetic acid (98%), 3-(methylphosphinico)propionic acid (98%), and iminodiacetic acid were obtained from Sigma (Steinheim, Germany). Labeled ^13^C_2_-^15^N-glyphosate, *N*-nitroso glyphosate, *N*-acetyl glyphosate, and *N*-acetyl AMPA were bought from TRC/BIOZOL (Echingen, Germany), ^13^C-^15^N-D_2_-AMPA from LGC Standards (Wesel, Germany), phosphonic acid from HPC Standard (Cunnersdorf, Germany), and trifluoroacetic acid (99%) from VWR (Darmstadt, Germany). The low concentration standard tune mix, purine, and HP-921 were from Agilent Technologies (Waldbronn, Germany). The OHNOON solution for the capillary coatings was prepared as described elsewhere [64]. Water from a purification system from ELGA LabWater (Celle, Germany) was used.

### Samples and sample preparation

Aqueous stock solutions of GLP, ^13^C_2_-^15^N-glyphosate ([M-H]^−^*m*/*z* 171.010, referred to as GLP171), AMPA, and ^13^C-^15^N-D_2_-AMPA ([M-H]^−^*m*/*z* 114.001, referred to as AMPA114) were prepared at a concentration of 1 g/L (5.92, 5.81, 9.01, 8.69 mmol/L). GLP and AMPA stock solutions were diluted to obtain concentrations of 3 and 1 mg/L, and 700, 500, 200, 100, 70, 50, 30, 10, 5 and 2 μg/L. For calibration in beer matrix, a mixture was used of GLP171 and AMPA114 with 4 and 10 mg/L, respectively. For GLP quantification in different beer samples, aqueous stock solution of GLP171 was diluted with water to 2 mg/L. Aliquots of the stock solutions were stored at + 4 °C for maximum 6 months. Stock solutions of DFA, GLU, IDA, MCPA, 3-MPPA, NAA, NAG, NNG, oxamic acid, phosphonic acid, and TFA were prepared at a concentration of 1 g/L; a mixture of the former analytes plus GLP and AMPA was prepared with a concentration of 50 mg/L, and diluted to achieve injection solutions with final concentrations of 250 and 500 μg/L.

#### Samples of sorption experiments and toxicological studies

Supernatants of sorption experiments with GLP on Al_2_O_3_ particles were prepared with 0.5 mmol/L KCl and initial GLP concentrations between 2 and 8 mg/L, the solutions were injected directly without further treatment, and quantification was achieved by external calibration (10 to 2000 mg/L) [65]. Toxicological studies with *Daphnia magna* [66] were conducted in a medium made of 2 mmol/L CaCl_2_, 0.5 mmol/L MgSO_4_, 0.75 mmol/L NaHCO_3_, and 0.08 mmol/L KCl according to ISO 6341 [67]. Calibration was done in the range of 25 to 200 μg/L in one 25 and three 50 μg/L steps. The medium was provided by R. Triebskorn and D. Werner from the Institute of Evolution and Ecology in Tübingen.

#### Metal complexation

To investigate the influence of metal complexation on CE separations, aqueous stock solutions with a concentration of 10 mmol/L of divalent and trivalent cations were prepared for each salt (MnCl_2_, CuCl_2_, CaCl_2_, MgCl_2_, Zn(OAc)_2_, NiCl_2_, CdCl_2_, Pb(NO_3_)_2_, AlCl_3_, and FeCl_3_). The injection solution was prepared with a final concentration of 1 mmol/L cation salt and 200 μg/L of GLP and AMPA (1.2 and 1.8 μmol/L, respectively, pH 2–4). The molar ratio between the cation and GLP was 850:1, and between cation and AMPA 560:1. Before analysis, injection solutions were equilibrated for 12 h at room temperature.

#### Beer samples

Fourteen beer samples from 2016 (Pilsner and naturally cloudy breed, see Application to beer samples section) were purchased from local stores. Beer samples were degassed by sonication for 15 min; naturally cloudy beer beverages were additionally filtered with Chromafil Xtra PTFE-45/25 filters (Macherey-Nagel, Düren, Germany). Calibration in beer matrix was done with an organic beer sample (Fidelio) in a similar range as for aqueous calibration (2 μg/L to 3 mg/L), with additionally 200 μg/L of GLP171 and 500 μg/L of AMPA114 for quantification; to estimate the LOD for GLP using large injection volumes, calibration samples of 2 and 5 μg/L were used. For quantification with the internal standard method (ISM) for all other beer samples, 500 μL of beer was mixed with 10 μL of GLP171 stock solution to achieve a final isotope standard concentration in the injection solution of 39 μg/L. For quantification via standard addition, Hasseröder Premium Pils was spiked with 10, 20, and 30 μg/L GLP. The mixture solution of AMPA, DFA, GLP, GLU, IDA, MCPA, 3-MPPA, NAA, NAG, NNG, oxamic acid, phosphonic acid, and TFA containing each analyte at a concentration of 50 mg/L was used to spike an organic beer (Fidelio) at a final analyte concentration of 250 and 500 μg/L in the injection solution. All aqueous and real samples were stored at − 18 °C.

### Instrumentation

#### Capillary electrophoresis

A 7100 Agilent CE System was used for CE-MS analysis. Capillaries coated with polyvinyl alcohol (PVA) were obtained from Agilent (Waldbronn, Germany) with an i.d. of 50 μm and cut to a length of 65 cm. Bare fused silica capillaries were obtained from Polymicro Technologies (Phoenix, USA). For acidic and alkaline buffers, an aqueous FA solution was titrated with aqueous ammonia to achieve the desired pH and degassed by sonication for 5 min prior to analysis. Before first use, capillaries were flushed with isopropanol (10 min) and BGE (20 min). Between runs, the capillary was flushed for 5 min with BGE. For storage, the capillary was flushed with BGE, isopropanol, and air for 10 min each, and stored in dry conditions. The final BGE chosen for analysis was 175 mmol/L FA titrated to pH 2.8 with ammonia (final concentration of approximately 40 mmol/L ammonia). BGE was exchanged after 10 runs to keep a high migration time precision. Separations were conducted at 25 °C (inside the CE housing) using a voltage of − 30 kV. In order to reduce analyte migration times and ensure stable electrospray conditions, an optimized inlet pressure of 30 mbar was applied during CE-MS analysis.

Standards were injected as aqueous solutions in 5 mmol/L ammonium formate buffer. Samples were injected at 75 mbar for 10 s (18 nL) if not stated otherwise. Large volume injection (LVI) with aqueous standards was done with 75 mbar for 40 s (71 nL), for beer beverages with 100 mbar for 40 s (94 nL). Analyte injection was followed by dipping the capillary into two extra vials filled with BGE to avoid analyte carryover between consecutive runs. The transfer of the method developed using aqueous standards to the analysis of samples with complex matrices required several adaptations. (1) We had to use a voltage ramp, starting at − 15 kV, decreasing within 2 min to − 30 kV to account for the high conductivity of some of the samples: at the beginning of the separation, this high conductivity zone evoked (a) high electric field strength in the BGE zone and thus run failures or (b) high current alarms and voltage re-adjustment, resulting in migration time shifts. (2) After sample injection, a plug of running buffer was injected at 100 mbar for 5 s to avoid sample components diffusing into the running buffer vial. (3) To further avoid carry over effects, the electrode was washed with BGE after each run using the “wash inlet electrode” command of the CE software.

#### Mass spectrometry

An Agilent 6550 iFunnel Q-ToF-MS instrument (Agilent Technologies, Santa Clara, CA) was coupled to the CE. A coaxial sheath liquid electrospray interface from Agilent Technologies (Waldbronn, Germany) and a Dual-ESI ionization source were used with an electrospray needle consisting of 80% platinum and 20% iridium (Agilent Technologies, Waldbronn, Germany). To improve the resulting electrospray, the needle geometry was optimized (compare section Platinum-iridium electrospray ionization needle). The sheath liquid (final method with a 50:50 (v/v) mixture of isopropanol:water containing 0.01% FA) was degassed upon ultrasonication and delivered by an isocratic 1260 infinity pump (Agilent Technologies, Waldbronn, Germany) at a flow rate of 5 μL/min with a split ratio of 1:100. For online recalibration during CE-MS analysis, the sheath liquid contained 0.2 μmol/L purine and 0.1 μmol/L HP-921 (both from Agilent Technologies; for HP-921, adduct with FA (*m*/*z* 966.001) was the reference). MS instrument parameters: Drying gas was delivered at 11 L/min at 150 °C, and nebulizer pressure was set to 5 psi during measurements. During preconditioning, injection and the first 6 s of the measurement, the nebulizer pressure was lowered to 3 psi (reduced suction effects during vial handling). The acquisition rate was 2 Hz; *m*/*z* range 50–1700; fragmentor voltage 380 V; electrospray voltage 4000 V. MS calibration was performed with the low concentration Tune Mix from Agilent Technologies.

#### Data processing

EICs were extracted and evaluated from mass profiles with a mass accuracy of 10 ppm using Mass Hunter Qualitative Software. Calibration curves in aqueous solution and beer matrix were evaluated from mass centroids with a mass accuracy of 20 ppm using MassHunter Quantitative Software, the linear range was determined by the signal areas, matrix effects were expressed by (sensitivity matrix)/(sensitivity aqueous solution) × 100% (with the sensitivity being the slope of calibration curve (signal area vs. spiked concentration)), and recovery in aqueous solution and beer matrix by (calculated concentration)/(spiked concentration) × 100%, LODs (signal to noise ratio SNR = 3) were estimated based on the SNR (mass profiles, 10 ppm, Qualitative Software) at the lowest calibration concentration, in case of AMPA in beer matrix at 50 μg/L. All figures were created with Origin 9.1.0G (OriginLab Corporation, Northampton, USA).

## Results

### Strategies to prevent glyphosate adsorption to the capillary wall

Strong tailing of GLP signals on bare fused silica capillaries was observed previously [39] and in our study using BGEs at pH 2 to 9. GLP was detected as a broad signal over several minutes. In later runs, we even failed to detect it, presumably due to irreversible binding and changes of the electroosmotic flow. On the first glimpse, adsorption may seem unlikely due to ionic repulsion at high pH and the almost neutral silica surface at low pH. However, from sorption studies, it is known that GLP predominantly binds to oxidic soil minerals via hydrogen bonding with its phosphonate group [7, 68–72]. In some cases, the carboxylic acid group is also involved in the binding event. The adsorption thus evokes inner sphere complexes, mostly with five- or six-membered rings with relatively high binding constants.

Strategies against GLP adsorption in capillary electrophoresis were summarized by Gauglitz et al. [11] and may include derivatization, the use of phosphate-based BGEs (e.g., Chui et al. [62]), working at elevated pH [33, 37, 42], extremely low pH [40], reduction of hydrogen bonding by shielding the capillary surface using dynamic [29, 34, 73] or permanent [39, 41, 43, 50] coatings. Also other separation modes, mostly MEKC, were used, most of them not compatible with MS detection. In our work using bare fused silica capillaries, peak shapes were not acceptable in ammonium acetate–based BGEs at any pH. For analysis by electromigration techniques, the low charge states of AMPA at low pH have to be taken into account, which led to a detection close to or with neutral substances. At higher pH, resolution was lowered (as also observed by Vidal [43]) and the overall selectivity for a direct method without derivatization was low as many organic acids are charged at this pH regime and may thus impair the analysis or necessitate further sample pretreatment. For our study, we decided to use permanent capillary coatings (both electrostatically adsorbed and covalently coupled) and a BGE of low pH for analysis. A cationic OHNOON coating was tested without success; strong interaction between the coating and GLP and AMPA was observed, even with effects from open tubular capillary chromatography (details can be found in the Electronic Supplementary Material (ESM) and in Fig. S1).

**Fig. 1:**
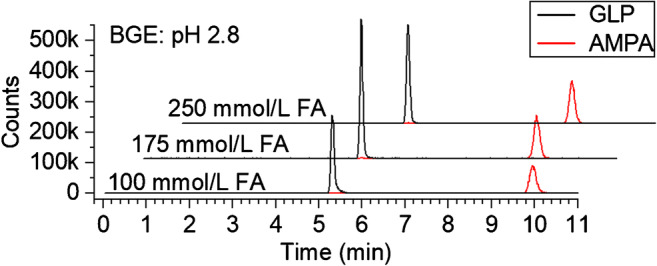
EICs of GLP and AMPA (1.69 and 1.11 mg/L (each 10 μmol/L), respectively) separated on a PVA-coated capillary (length 65 cm, i.d. 50 μm) using BGEs of ammonium formate at pH 2.8 (FA titrated with aqueous ammonia) with different ionic strength (see figure legend). Injection was accomplished at 50 mbar for 5 s, separation voltage was − 30 kV, 50 mbar pressure were applied. Sheath liquid was isopropanol:water 1:1 with additional 0.1% FA, flow rate 0.5 mL/min (1:100 split)

A neutral PVA-coated capillary exclusively presents non-acidic hydroxyl groups to the electrolyte solution; hence, interaction with the phosphonate group was low at acidic pH. As visible from Fig. 1 good peak shapes, resolution and separation efficiency with plate numbers of up to 85,000 (130,000/m) were achieved for the analytes under optimized conditions (175 mmol/L FA titrated to pH 2.8 using approximately 40 mmol/L ammonia). However, different capillary batches significantly differed in performance regarding migration time stability and signal shape, possibly due to an aged PVA coating (data not shown). We observed that the optimized BGE made of formic acid and ammonia was also suitable for separation on bare fused silica capillaries (which was not possible using acetic acid–based BGEs), when the samples contained significant amounts of phosphate, as present, e.g., in soil extracts, which competes with sorption sites [74]. However, the use of PVA-coated capillaries is required when samples contain proteins, since protein sorption onto the bare fused silica capillary inner surface can hardly be avoided [75]. Distinct matrix effects and possible effects by sample-induced transient ITP will have to be studied for such samples.

### Platinum-iridium electrospray ionization needle

A standard stainless steel ESI needle used in negative MS polarity and reverse CE polarity leads to strong corrosion of the electrospray needle, accompanied by the migration of metal cations into the separation capillary, which may even impair the separation [76]. For reliable CE-MS analysis of anionic compounds, the use of a platinum-iridium needle is necessary [76]. However, the commercially available platinum-iridium ESI needle (Agilent Technologies, Waldbronn, Germany) has a cylindrical tip geometry, which lacks coaxial focusing of the nitrogen gas flow at the sprayer tip. Increased migration times compared with the iron needle were observed under identical separation conditions due to lower suction effects. A tapering step as common for steel ESI needles by short electrochemical polishing to decrease the tip wall thickness was not successful. Finally, the performance of the platinum-iridium electrospray needle was optimized with regard to ESI stability (TIC noise RSD < 10%), signal intensity, and migration time precision by reducing the wall thickness via grinding and mechanical polishing of the needle tip (by goldsmith Ulrich Wehpke, Krefeld, Germany). Different geometries of the platinum-iridium needle tip are shown in ESM Fig. S2.

### Method optimization

pH and ionic strength of the BGE were optimized using PVA-coated capillaries. For method optimization, we chose common MS-compatible BGEs made of FA and ammonia. Method optimization focused on separation efficiency, signal shapes as well as migration time precision as indicators for reduced adsorption phenomena, and the overall analysis time. For BGE optimization, an additional pressure of 50 mbar was applied during separation; for the optimized method, the pressure was set to 30 mbar as a compromise between stable separation and ESI conditions and minimized signal broadening due to the parabolic flow profile.

#### pH of the BGE

In principle, fast analysis is possible at basic pH due to the high analyte charge. However, we were not able to establish stable separation conditions at pH 9.2. Increasing peak asymmetry and peak broadening (full width at half maximum increased from 0.12 to 0.25 min) and migration time shifts (RSD > 10%, *n* = 10) unveiled pronounced interaction between GLP and the PVA capillary surface at high pH. In contrast, peak widths and migration times for AMPA remained constant. Using 100 mmol/L FA titrated with ammonia to pH 3.6, peak area precision and migration time stability were very good for GLP. For AMPA, however, peak tailing and low migration time precision were observed (see ESM Fig. S3).

Using 100 mmol/L FA titrated to pH 2.8, peak area precision and resolution were good for all analytes. Separation efficiency was higher than for any other pH value tested, but for GLP, a slight tailing was observed (see Fig. 1a). It has to be noted that AMPA is neutral at this pH (anionic only at pH > 4), so it is transported only by EOF and the additional pressure applied during separation. For complex samples, AMPA quantification is impaired by the presence of neutral matrix components. We accepted this drawback as our major focus was on GLP analysis and as the use of an isotopically labeled AMPA standard enabled its quantitative analysis (see below).

#### Ionic strength

In preliminary experiments, we observed that the ionic strength was important to achieve a high separation efficiency and higher migration time precision (which was low when low concentrations of FA were used without ammonia to reach the desired pH). In order to further optimize separation efficiency and signal shape, especially for GLP, BGEs with elevated concentrations of 175 mmol/L and 250 mmol/L FA titrated to pH 2.8 using ammonia were investigated. The BGE with 175 mmol/L FA revealed improved peak shapes due to the higher ionic strength (see electropherograms in Fig. 1), and up to 20% higher GLP signal areas compared with 250 mmol/L FA. Additionally, using 250 mmol/L FA, the separation voltage was automatically reduced within the first 3 min of separation, to keep the separation current below 40 μA avoiding Joule heating. With increasing ionic strength, the separation current declined more extensively due to the counter ion of the BGE (ammonium ions) being gradually replaced by protons from the sheath liquid upon electromigration to the inlet [77]. Accordingly, the pH was reduced during the run depending on the initial ammonium concentration in the BGE. This phenomenon can hardly be avoided. pH steps will be present in most CE-MS methods, even when BGE and sheath liquid contain the same ions and only concentration differences are present [78]. We used 175 mmol/L FA with 40 mmol/L ammonia as final buffer composition giving best signal shapes, responses, and reduced Joule heating. Just flushing the capillary with BGE between runs was sufficient to maintain high precision (migration times RSD < 0.4, peak area RSD < 3, *n* = 10); for real samples the electrode was additionally washed to avoid matrix carry over into the run buffer vial (see below).

#### Optimization of sheath liquid composition

The isopropanol:water ratio of the sheath liquid was varied in the range of 35–65% (v/v), flow rates between 0.5 and 0.65 mL/min, and a nebulizer pressure in the range of 3–6 psig. In contrast to many other studies (e.g., ref. [79]), we did not find a pronounced impact of the solvent:water ratio on GLP ionization efficiency, as long as the water content was kept at or below 50%. Similarly, little influence of the sheath liquid flow rate and nebulizer pressure was observed, pointing to a high robustness of the ESI process for GLP ionization. With 35% water in the sheath liquid, the separation current fluctuated sinusoidally by 10% during the run, occasionally accompanied by electrical contact loss and interruption of the measurement. For further measurements, an isopropanol:water ratio of 1:1 and a nebulizer pressure of 1.36 bar (5 psig) were used.

Principally, low concentrations of formic or acetic acid in the sheath liquid of 0.1–1.0% guarantee adequate conductivity of the sheath liquid [80]. However, we observed pronounced ion suppression at these concentrations, corroborating findings for LC-MS analysis with eluents with a high FA concentration [81]. Decreasing the FA concentration from 0.1 to 0.01% increased signal intensity by 60%. Highest signal intensities were observed at 0% FA in the sheath liquid, however, at the cost of a reduced electrospray stability, so 0.01% FA (2.6 mmol/L) was used as a compromise. Using alkaline sheath liquid conditions with 0.02 or 0.5% ammonium hydroxide (2 and 50 mmol/L), ionization efficiency of GLP decreased by 20 and > 90%. In addition, migration of counterions, presumably ammonium ions from the SL into the capillary, was observed (for discussion, see ESM Section D with Fig. S4 and [82]), accompanied by reduced migration times by 12 to 22% and lower migration time precision (see Ionic strength section).

#### Online preconcentration

Only few online preconcentration methods are suitable for samples of elevated ionic strength [83]. For beer samples, sample-induced transient isotachophoresis (sITP) was possible with (in)organic ions present as ionic macrocomponents [84, 85] with suitable electrophoretic mobilities.

##### Aqueous standards

In order to improve detection limits, LVI was investigated with injection volumes of 18–89 nL (injection at 75 mbar for 10 to 50 s) using an aqueous GLP solution of 10 μg/L (59 nM). At high injection conditions (75 mbar for 40 s, 71 nL and thus 4 times higher than without LVI), LOD was lowered by a factor of 3 with acceptable peak broadening. AMPA is neutral at pH 2.8 and band broadening was significant (20 to 70 s base width); the LOD was not improved.

##### Beer samples

**Fig. 2:**
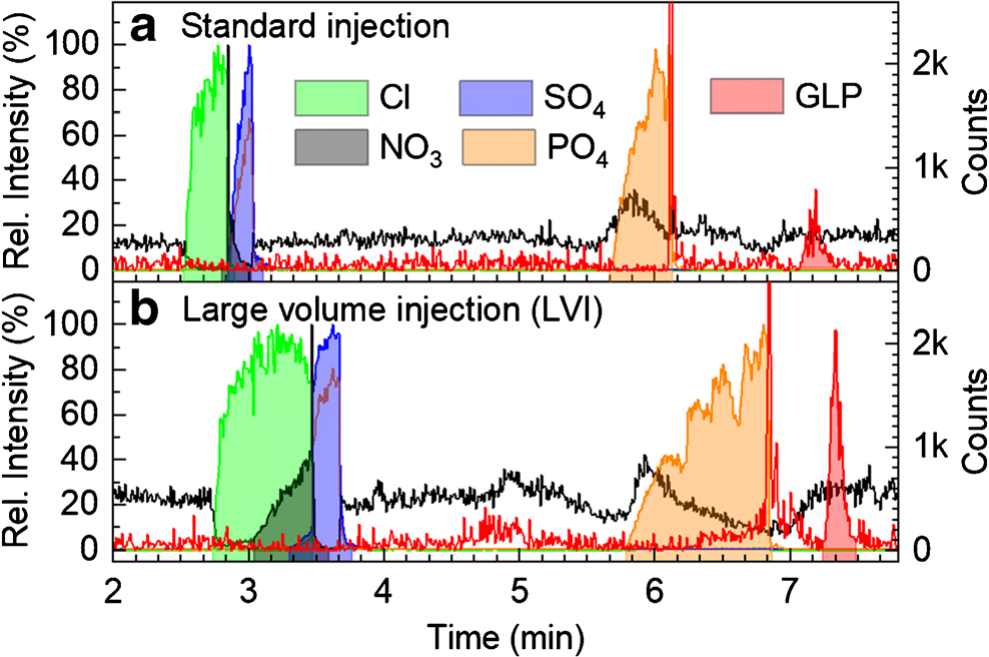
sITP in CE-MS electropherograms of beer samples with EICs of anions in relative intensity (left axis), EIC of GLP in counts (right axis) in Wicküler Pilsner beer originally containing ca.14 μg/L GLP for **a** standard injection at 75 mbar for 10 s (18 nL) and **b** LVI at 100 mbar for 40 s (95 nL). Separation was achieved on a PVA-coated capillary (i.d. 50 μm, length 65 cm) using a BGE with 175 mmol/L FA titrated to pH 2.8 with ammonia, and a separation voltage of − 30 kV with additional 30 mbar pressure. Within the first 2 min of separation, the voltage was ramped from − 15to − 30 kV. Sheath liquid was isopropanol:water 1:1 with additional 0.01% FA

LVI combined with sITP using natural ionic macrocomponents of the samples as transient leaders was tested as preconcentration method for organic beer samples spiked with 2 or 5 μg/L GLP and injection volumes of 70–118 nL (100 mbar for 30–50 s). Comparing standard injection (Fig. 2a, 75 mbar for 10 s, 18 nL) and LVI (Fig. 2b, 100 mbar for 40 s, 95 nL), large signals of inorganic salts were observed as well as a moderate migration time shift for GLP from 6 to 6.5 min. Obviously, sITP was evoked by the sample components chloride, nitrate, sulfate, and phosphate acting as transient leaders (in order of decreasing electrophoretic mobility), its relevance depends on its concentration. At LVI conditions, chloride, nitrate, and sulfate were still stacked isotachophoretically upon detection. Destacking was proven to start at the front boundary (MS direction) of the sITP stack (data not shown). At too high injection volumes, the capillary length was too short to fully resolve the sITP stack and GLP was stacked behind phosphate.

Experiments with doubly deuterated FA indicated that FA has an electrophoretic mobility suitable to be a transient terminator only for GLP. The extent of sITP depends on the concentration of the transient leaders. In beer samples, the most critical macrocomponent was phosphate (phosphorous content in beer in the range of 0.3–15 mmol/L [86]). Using disodium phosphate at a concentration of 75–100 mM for GLP extraction from soil, the transient ITP did not fully resolve and GLP was detected in the isotachophoretic stack (data shown elsewhere [82]). At the highest injection volume, poor resolution between GLP and phosphate led to quenching of the GLP signal; poor resolution between GLP and a near isobaric matrix compound (*m*/*z* 167.996) impaired accurate quantification (see ESM Fig. S5); in addition, co-migration of a matrix compound near isobaric to GLP171 was also observed. When using the Q-ToF in MS/MS mode, reduced sensitivity due to ion losses during fragmentation was observed. Thus, injection conditions had to be limited to 100 mbar and 40 s to assure baseline separation of GLP and these matrix compounds.

#### Influence of divalent cations on the separation of GLP

Di- and trivalent metal cations were added to the sample to investigate influences on CE separations at molar ratios between cations and GLP of 850:1 (see Samples and sample preparation section). The electropherograms from injection solutions with *divalent* cations (Mn^2+^, Cu^2+^, Ca^2+^, Mg^2+^, Zn^2+^, Ni^2+^, Cd^2+^, Pb^2+^) showed a slight increase in migration times of 5–10 s compared with sample solutions void of these cations. This is caused either by increased conductivity and thereby reduced electric field strength within the sample plug [87], by transient ITP phenomena, or by a relatively fast (compared with the analysis time) on-capillary dissociation of GLP-metal complexes at low pH [88]. However, these increased migration times proved to be stable (RSD < 0.3%). The signal area differences were within the quantitative precision and thus comparable for different cation solutions (RSDs < 6%, *n* = 3 within one run series, over all run series RSD = 5%, *n* = 9). This low influence of metal cations in CE is presumably due to the high lability of these complexes in the BGE of pH 2.8: when GLP-metal complexes (which would be neutral or positively charged) are present but dissociate quickly at the beginning of the run, GLP and the cations migrate in opposite direction, hindering complex reformation. We did not observe signal broadening so that we assume very fast dissociation kinetics; high GLP signal areas of 96–107% compared with sample void of cations were obtained.

In contrast, the GLP complexes of *trivalent* Al^3+^ and Fe^3+^ proved to be relatively inert in the separation buffer and impaired separation; the recovery of GLP from Al-containing solutions was only 49% at a molar ratio of 850:1. We observed a strong broadening of the GLP signal with peak base widths over 1 min. We were able to reduce but not fully overcome this matrix effect by adding 50 mmol/L Na_2_HPO_4_ to the sample, which leads to precipitation of insoluble aluminum or iron phosphates. The addition of 20 mmol/L EDTA (common for derivatization in LC [21]) to the injection solution spiked with Al^3+^ did not improve the separation; instead, a dynamic equilibrium was induced between GLP-Al, EDTA-Al, and GLP-EDTA-Al complex species, which were poorly separated but could be discriminated with MS (data not shown). In contrast to our results for CE-MS, the presence of Al^3+^ and Fe^3+^ did not impair RPLC-ESI-MS, but strong retention time shifts were observed for divalent Cu, Zn, or Mn [22]. These differences may in part be explained by the different pH values used for the separation (pH 9 for RPLC-MS [22] and 2.8 for our CE-MS method).

### Method performance

#### Separation selectivity and matrix tolerance

The broad applicability of the developed method is exemplified by the analysis of GLP in various samples differing in their matrix. GLP was quantified in aqueous solutions of sorption experiments to Al_2_O_3_ nanoparticles as a model of soil minerals and in the exposure medium for *Daphnia magna* during a toxicological study. Beer beverages represent a complex matrix containing many organic acids. The conductivity of the BGE (175 mmol/L FA titrated to pH 2.8 using ammonia) was 2.7 mS/cm, of the exposure medium 0.6 mS/cm, and of beer beverages around 1.8 mS/cm. Hence, field-amplified sample stacking can be expected for the aqueous samples (sorption and *Daphnia* medium) but not for beer samples.

**Fig. 3:**
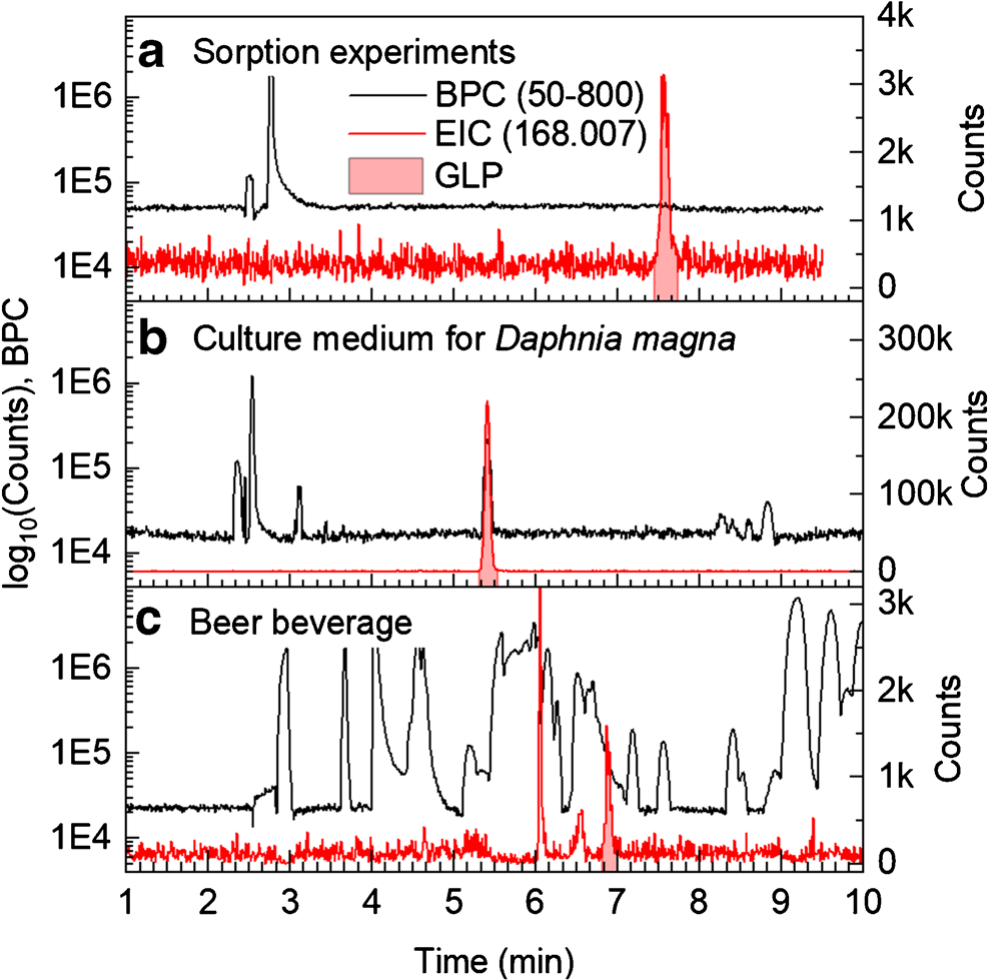
Base peak current (*m*/*z*-range 50–800, excluded masses are reference masses, sulfate, phosphate, and clusters thereof) in logarithmic scale (left axis) and EIC of GLP (*m*/*z* 168.007) with shaded GLP signal (right axis). The samples were from **a** aqueous sorption studies on Al_2_O_3_ nanoparticles with 0.5 mmol/L KCl, **b** toxicological studies in culture medium (additional 50 mbar pressure during separation), and **c** a German beer (Hasseröder Pils). GLP concentrations were determined to be **a** 18.6 μg/L, **b** 1 mg/L, and **c** 14.4 μg/L. Injection was done hydrodynamically at 75 mbar for 10 s. Further separation conditions as in Fig. 2

Representative base peak currents (BPC) and extracted ion currents (EIC) of GLP (*m*/*z* 168.007) for the three sample types are shown in Fig. 3. For all samples, separation of GLP from most matrix components was achieved within 5–8 min. Sorption experiment and *Daphnia* medium samples (Fig. 3a and b) showed relatively clean electropherograms with mainly chloride and sulfate visible. The signals at 8–9 min of the *Daphnia* medium were from organic components. In contrast, the BPC of beer samples (Fig. 3c) revealed many large signals over the whole electropherogram; inorganic salts and organic acids are present. Various organic matrix components (migration time range 3.5–6 min) and inorganic anions (compare Fig. 2) migrated faster than GLP and were well separated from the analyte. A matrix component with *m*/*z* 361.200 and possibly also organic phosphates migrated directly in front of GLP; tailing or incomplete stacking of these matrix components probably influenced ionization efficiency, but also GLP preconcentration by sITP. After 8 min, mostly small organic acids (not further identified) were observed, which were present in beer in relatively large quantities [89]. Overall, only strong acids can be expected as most organic carboxylic acids (fatty or amino acids) are neutral or positively charged at the BGE pH of 2.8, giving rise to a high matrix tolerance of the method. Due to the Q-TOF’s high mass accuracy, only two potentially interfering matrix components both with *m*/*z* 167.996 were observed migrating close to GLP at 7 min (Fig. 3c). With standard volume injection, resolution between GLP and the closer co-migrating near isobaric compound in different beer beverages was 2.4–2.6, so that no interference is expected, while for LVI with 100 mbar for 40 s, they were just baseline separated. Theoretical plate numbers are 20,000–35,000 for standard injection (depending on the matrix load) and 17,000 for LVI. Since AMPA is neutral and migrates close to the EOF, distinct matrix effects were observed (see Quantitative aspects, linear range, and limits of detection section).

#### Long-term stability and precision

Using a single PVA capillary, we were able to perform more than 200 measurements of standards and matrix-loaded samples without loss in separation performance and peak shape. Precision for standards was calculated for peak area to be 2–10% RSD and for migration time 0.3–0.8% RSD (*n* = 9). Intermediate precision (5 days) for migration time and peak area was 6.4% and 17.5%, respectively (*n* = 36). This confirms the suitability of neutrally coated PVA capillaries for GLP analysis.

With regard to precision analyzing real samples, absolute changes in migration times (see Fig. 3) were due to sITP phenomena, differences in applied pressure during separation (30 vs. 50 mbar), and the use of the voltage ramp in the case of beer samples. Migration times for a specific sample were highly reproducible with < 0.7% RSD (for the most complex beer samples even < 0.3% RSD). Signal shape was fair, while at high concentrations of GLP, a slight tailing was observed. RSDs of signal areas of GLP and AMPA in aqueous samples were in the range of 1–11%, but usually below 10% RSD, in beer matrix in the range 1–9%, while for AMPA, sometimes higher values, but below 15% RSD, were observed (see Table S1 in ESM).

#### Quantitative aspects, linear range, and limits of detection

Three different methods for quantification were tested: external calibration with aqueous standards, standard addition, and internal standard method (ISM). Quantification via external calibration can only be applied, if matrix effects are negligible. Due to the CE method’s high selectivity, this was the case for samples from sorption experiments and toxicological studies with *Daphnia magna* (both mainly containing inorganic salts). For beer samples, matrix effects (co-migrating compounds with high signal intensities) impaired ionization efficiency of GLP, so that standard addition or quantification via ISM had to be used. Matrix-matched calibration would not be an option, since matrix composition of similar beer types can still be different, and blank samples of the same beer are most likely not available. Quantitative precision for GLP in beer matrix was high even when the concentration was close to the LOD. The RSD of concentrations determined via ISM ranged between 4 and 11% (*n* = 3). Comparing ISM and standard addition (10, 20, and 30 μg/L GLP were spiked to the sample), the calculated GLP contaminations of Hasseröder Premium Pils were 14.4 and 13.2 μg/L, respectively. Hence, both quantification methods showed good accuracy for GLP analysis. As ISM is more efficient for larger sample numbers and accounts for temporal changes in ESI conditions, we chose ISM using GLP171 for further analysis.

The linear range for aqueous samples, *Daphnia* medium, and beer matrix (Fidelio) was determined in triplicate for GLP and AMPA via a calibration curve with standard injection. In general, GLP contamination of beer was below 40 μg/L (see Application to beer samples section); AMPA was never detected. Calibration curves for GLP and AMPA showed good linearity within the calibrated range; however, distinct matrix effects were observed in beer samples, especially for AMPA: the higher LOD for AMPA in beer samples compared with aqueous standards (30.6 vs. 3.3 μg/L, respectively) is due to AMPA being neutral at the pH chosen, so that co-migration with neutral matrix components and thus ion suppression occurs, which reduces the sensitivity to below 9% compared with aqueous samples. Ionic matrix components proved to be advantageous for GLP preconcentration as they acted as transient leaders in sITP, which could still be well separated in LVI. In contrast, the LOD for AMPA in beer matrix was not significantly improved using LVI; however, the signal shape improved compared with the aqueous standard measurements. We assume that this is due to sITP preconcentration occurring in the sample plug having a pH high enough to have AMPA charged. All validation parameters are summarized in Table 1.

**Table 1:** Linear range (*n* = 3) (correlation coefficient *R*^2^ with error weight 1/*x*^2^) and LODs for GLP and AMPA in aqueous samples, *Daphnia* medium (see Samples and sample preparation section), and organic beer sample (Fidelio). If large volume injection (LVI) was tested, values are given in brackets

Sample	GLP					AMPA				
	Linear range (μg/L)	*R*^2^	Matrix effect	Accuracy	LOD (μg/L)	Linear range (μg/L)	*R*^2^	Matrix effect	Accuracy	LOD (μg/L)
Aqueous standard	5–3000	0.9996		95.5–105.0%	4.2 (1.4)	10–3000	0.9957		87.2–99.5%	3.3
*Daphnia* medium	25–200	0.9967	–	–	4.7	25–200	0.9938	–	–	6.2
Beer beverage	10–3000	0.9912	93%	94.3–110.7%	5.3 (2.1)	50–3000	0.9723	8.7%	80.2–100.4%	30.6

#### Application to beer samples

The developed method was applied to the analysis of 14 beer beverages using ISM. Prior to analysis, all samples were degassed by sonication for 15 min to avoid CO_2_ release inside the capillary. The pH of the degassed samples was around 4. All beer samples were spiked with 40 μg/L of ^13^C_2_-^15^N-glyphosate (GLP171) solution. If the detected GLP concentration was close to or below the LOD, LVI was used. Quantitative results are summarized in Table 2; a representative electropherogram is shown in Fig. 3c.

**Table 2:** GLP residues determined with CE-MS via ISM with RSD values of quantified concentration (*n* = 3) from different beers brewed in Germany and geological latitude of the brewery. Beers were spiked with 40 μg/L of GLP171. n.d., not detected

Beer beverage	[GLP] (μg/L)		RSD (%)	Latitude (North)
Astra Rotlicht	35.1	± 3.1	8.7	53.0°
Tuborg Pilsner	33.9	± 2.9	8.5	53.0°
Hasseröder Premium Pils	14.1	± 1.4	10.2	51.8°
Wicküler Pilsner	13.6	± 0.8	6.1	51.5°
Jever Fun (alcohol-free)	12.7	± 1.4	10.9	53.6°
Carlsberg Pilsner	3.4	± 0.1	4.2	53.0°
Alpirsbacher Spezial	n.d.		–	48.4°
Fidelio (organic)	n.d.		–	47.8°
Kloster Landbier	n.d.		–	48.5°
Gold Ochsen Original	n.d.		–	48.4
Riegeler Landbier	n.d.		–	48.0°
Rothaus Tannenzäpfle	n.d.		–	47.8°
Stuttgarter Hofbräu Bügel Spezial	n.d.		–	48.8°
Wulle Vollbier hell	n.d.		–	48.8°

Six out of 14 beer samples were tested positive for GLP with contaminations ranging from 3.4 to 35 μg/L. In Southern German beers, the concentration of GLP was below the LOD. AMPA was not detected in any of the investigated samples. This is possibly due to the high load of neutral matrix components, possibly quenching AMPA signals and deteriorating the LOD to around 30 μg/L.

#### Applicability of the method to other anionic contaminants

The high selectivity of a separation BGE of low pH for strong acids has been shown for other applications such as glucosinolates in *A. thaliana* seeds [90], and sulfates, sulfonates, and phosphates in urine [91]. We here want to show the principal applicability of this strategy to other environmentally relevant strong acids using the method optimized for glyphosate: *N*-nitroso glyphosate (NNG) is a byproduct of GLP synthesis [92]. *N*-Acetyl glyphosate (NAG) and *N*-acetyl AMPA (NAA) are relevant GLP metabolites in modified crops containing *gat* gene [93]. Glufosinate (GLU) is a herbicide structurally related to GLP; a degradation product of GLU is 3-(methylphosphinico)propionic acid (3-MPPA) [94]. MCPA (2-methyl-4-chlorophenoxyacetic acid) is a herbicide with converse properties to GLP; it poorly adsorbs to soil components and thus is highly mobile in the environment [95]. Trifluoroacetic acid (TFA) is a persistent, highly water soluble industrial chemical, which is also derived by (photo)degradation from refrigerants, (per)fluorinated chemicals, pharmaceuticals, and pesticides [96]. Difluoroacetic acid (DFA) is probably mostly derived from TFA and has not yet been considered in the environment before [97]. Oxamic acid is an oxidation product formed upon ozonation during drinking water treatment, most likely derived from N-containing organic matter [98]. Iminodiacetic acid (IDA) is a chelating agent (e.g., immobilized for ion exchange resin) used for medical diagnosis [99], and structurally related to GLP, but its phosphonic acid moiety is replaced by a carboxylic acid moiety. Phosphonic acid acts as a fungicide, e.g., to control downy mildew in vineyards [100].

**Fig. 4:**
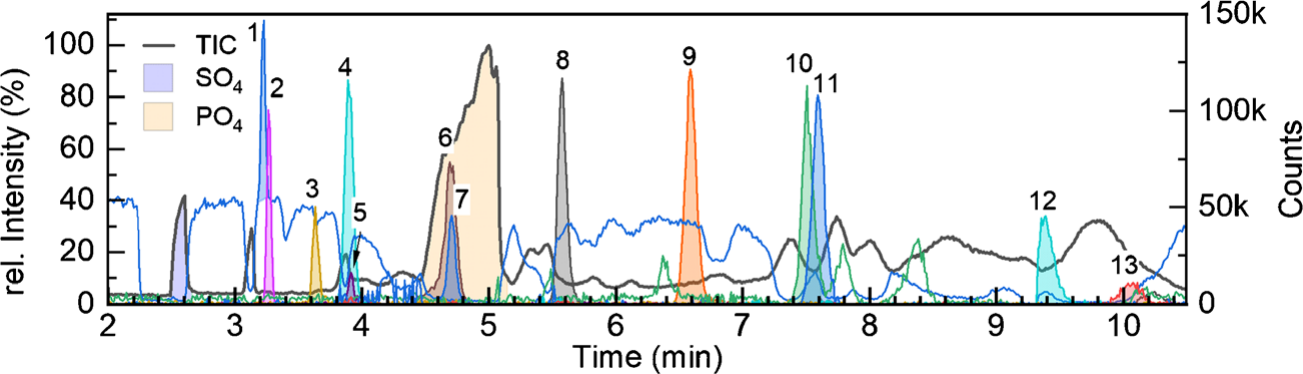
Different pesticides and environmentally relevant pollutants spiked at a concentration of 250 μg/L to an organic beer sample (Fidelio). Total ion current and highlighted signals of sulfate and phosphate (left axis). Electropherograms of 13 different analytes (left axis): (1) TFA, (2) DFA, (3) phosphonic acid, (4) NNG, (5) oxamic acid, (6) NAG, (7) NAA, (8) GLP, (9) 3-MPPA, (10) IDA, (11) MCPA, (12) GLU, and (13) AMPA. Intensities of TFA and DFA (1, 2) are divided by factor 5; and of phosphonic acid, oxamic acid, IDA, MCPA, GLU and AMPA (3, 5, 10–13) are multiplied by factor 3 to fit the axis range. Separation was conducted on a PVA-coated capillary with a total length of 60 cm, all other parameters as in Fig. 3c

Most of the analytes were baseline separated, as indicated by resolution for two successively migrating compounds (see Table 3). Electropherograms of the analytes are shown in Fig. 4. TFA and DFA are badly separated, probably due to similar hydrodynamic radii. The pairs of NNG/oxamic acid and NAG/NAA are co-migrating. For all compounds, the LOD in aqueous solution was below 10 μg/L (based on calculation of SNR at 250 μg/L), except for oxamic acid and TFA. The relatively high LOD for TFA (especially compared with DFA) is due to high noise, caused by the used fluorinated mass calibrant for online *m*/*z* calibration. In the case of beer matrix, the LODs deteriorated with increasing migration time due to co-migration of weak organic acids originating from the beer matrix (this is the case for IDA, MCPA, GLU, and AMPA), or due to co-migration of phosphate and phosphonate derivatives, resulting in analyte signal quenching in case of NAA. IDA, MCPA, GLU, and AMPA are co-migrating with matrix constituents of low or no charge and suffer from quenching by having 6–56% of signal intensity compared with aqueous standard.

**Table 3:** Different anionic analytes listed in their order of migration in organic beer matrix (Fidelio). Resolution of neighboring analytes, estimated LODs based on SNR, RSD of signal area (*n* = 3), and matrix effects (expressed as ratio between (signal area in matrix)/(signal area in aqueous solution) × 100%) were obtained from spiked concentrations of 250 μg/L. Experimental parameters as in Fig. 4

	Analyte	Resolution		LOD (μg/L)		RSD signal area (%)		Matrix effect (%)
		Water	Beer	Water	Beer	Water	Beer	
1	TFA	0.8	0.6	66.0	53.3	7.4	2.6	112.7
2	DFA	6.4	4.9	2.8	2.7	8.3	0.4	102.0
3	Phosphonic acid	3.1	2.4	30.4	28.5	10.6	0.5	93.7
4	NNG	0.0	0.2	2.9	3.7	11.5	2.8	115.3
5	Oxamic acid	4.4	6.3	53.7	90.7	9.3	15.4	58.6
6	NAG	0.1	0.1	1.8	4.3	11.5	3.6	46.2
7	NAA	8.3	6.9	2.7	15.3	7.8	1.7	32.0
8	GLP	6.1	6.8	2.7	6.2	6.1	3.5	84.5
9	3-MPPA	4.5	5.5	1.6	3.3	3.8	2.0	89.4
10	IDA	0.8	0.5	20.5	40.8	7.1	7.4	41.6
11	MCPA	6.5	8.9	9.2	14.3	7.1	3.6	55.8
12	GLU	2.2	2.9	6.8	39.9	3.1	1.7	23.8
13	AMPA			3.3	24.4	3.4	2.1	6.4

## Discussion

The properties of GLP are unfavorable for direct LC analysis. In contrast, its high charge over nearly the whole pH range (with an anionic charge down to pH 2.3) allowed us to develop a direct CE-MS method without derivatization. The high selectivity of our CE separation was due to a BGE of pH 2.8 and reverse polarity. Under these conditions, only strongly acidic organic compounds and mineral acids have sufficiently high electrophoretic mobilities to reach the MS. However, this advantage was on the cost of limits of detection for AMPA, which can only be quantified at concentration above 30.6 μg/L in beer matrix using an isotopically labeled internal standard. This is due to its co-migration with neutral matrix components. Mostly, neutral to alkaline pH BGEs were used by other groups [33–35, 39, 42, 43, 62]; only Safarpour and Asiaie [40], and Iwamuro [41] used acidic BGEs at pH 2.5 and 3.4 for GLP analysis, respectively. Safarpour and Asiaie [40] analyzed GLP contaminations in dried granule formulations within only 5 min separation time by CE-MS. Matrix effects due to anionic surfactants and salts possibly present in the formulations were not discussed. Iwamuro et al. [41] mixed green tea with a GLP formulation to have a final concentration of 820 mg/L GLP; no interferences by matrix components were observed at this high concentration and due to the fact that anions were separated by CE but detected in the positive ionization mode by ESI-MS. However, the LODs stated would not be sufficient for GLP quantification in beer and food samples. Goodwin et al. [39] used a capillary coated with linear polyacrylamide and a BGE of 1 mmol/L acetic acid in a water:methanol (50:50, v/v) mixture. With an injection volume of 70 nL, LODs of 169 μg/L for aqueous standards but 422 μg/L in wheat extracts were achieved using a sheathless CE-MS interface. In part, the relatively high detection limits compared with our study were due to poor peak shapes from the low ionic strength of the BGE and the high pressure of 2 psi (138 mbar) applied during separation, which was required by the sheathless ESI interface and absence of EOF.

We achieved LODs for GLP in aqueous samples and beer beverages of 1.4 and 2.1 μg/L, respectively. Of all derivatization-free CE methods surveyed [11], only Safarpour and Asiaie (CE-MS) [40], See et al. (CE-C^4^D) [34], and Horčičiak et al. (chip-based column coupling of ITP/CE-C^4^D) [31] achieved comparable or better LODs down to 0.1 μg/L for aqueous samples. However, the matrix tolerance and selectivity were low using conductivity detection [31, 34] and preconcentration methods such as large volume sample stacking or field-enhanced/amplified sample injection [34]. Besides sample preparation strategies, further improvements of LODs may be possible using different CE-MS interfaces. However, when using conditions of very low EOF, sheathless ESI-MS approaches such as presented by Goodwin et al. [39] for GLP analysis or the commercialized sheathless interface originally presented by Cao and Moini [101] are problematic as the low volume flow from the capillary has to be increased using pressure. In addition, the choice of the BGE is more limited as the requirements for MS compatibility are even higher than for sheath liquid interfaces. A compromise may be the sheath flow interface using electrokinetic pumping [102] or nanoESI [103]; however, its applicability to GLP still has to be shown.

Comparing our method to LC-MS-based approaches, matrix effects were low despite the negligible sample preparation with degassing only. Anastassiades et al. [18] tested five different LC columns (hypercarb, anion exchange, and HILIC) after matrix-adapted extraction with acidified methanol (“QuPPe”) from different commodities (among them grapes, barley, lentils, cucumber). LOQs of GLP in apple, barley, cucumber, and grape samples were 20 μg/kg. An application note by Sciex [104] demonstrated GLP analysis using a mixed-mode LC column with QTrap-MS using multiple reaction monitoring with retention times of underivatized AMPA and GLP of 1.2 and 2.2 min and LODs of 0.2 μg/L in beer beverages. However, the mass spectra clearly showed the limited selectivity, as GLP is poorly separated from several matrix components. As in our study, AMPA was not detected in beer. Nagatomi et al. [105] analyzed GLP and AMPA in beer beverages and barley extracts after enrichment and purification using anion exchange solid phase extraction. With this derivatization-free LC-MS/MS method, they achieved LOQs of 10 μg/kg together with a high accuracy and recovery; however, they obtained a base width of the GLP signal in standard solutions of almost 1 min at a retention time of 6 min.

The influence of a high concentration (up to 1 mmol/L) of divalent inorganic cations (such as Ca^2+^, Mg^2+^, Pb^2+^, see Samples and sample preparation and Influence of divalent cations on the separation of GLP sections) on the CE separation and MS detection proved negligible. However, trivalent Al^3+^ and Fe^3+^, present, e.g., in soil samples, evoked migration time shifts. Adding Na_2_HPO_4_ to the sample reduced this matrix effect. Together with the use of an isotopically labeled internal standard, identification and quantification of GLP is possible, especially with regard to the low concentrations of these metal cations normally present in (food) samples (e.g., iron in dried beans and peas < 1 mmol/kg [106]). In contrast, for derivatization and chromatographic separation, divalent cations, especially Ca^2+^ and Mg^2+^, were more problematic [20–22], as they occur in larger concentrations in many samples.

Our new CE method clearly has a simple instrumental setup compared with IC-MS, where an eluent generator and a suppressor become necessary. Similar LODs of 2.5 μg/L (IC-MS) [27] and < 2 μg/L (CE-MS) in samples were obtained. Matrix effects in CE-MS were low given the high separation selectivity. For IC, a two-dimensional method was discussed to reduce matrix effects, however, at the cost of analysis times [28].

For our study, it is interesting to note that GLP was only detected in German beer samples, where the brewery is located at a geodetic latitude > 50° North. Assuming that the brewery predominantly uses local barley sources, the reasons may in part be historic: succession rules favored large farms in the Northern regions of Germany. With the foundation of Agricultural Production Cooperatives (Landwirtschaftliche Produktionsgenossenschaft), the German Democratic Republic further fostered large agricultural units with accordingly large fields [107], for which non-tillage agricultural management (using GLP) may be advantageous [108] compared with the rather small agricultural units and fields in the South of Germany, where plowing dominates [2]. In addition, climate conditions favor GLP application in the more humid conditions present in the coastal areas, e.g., for desiccation [2].

The CE-MS method was not only applied for samples containing high salt and matrix loads but also for a variety of other strong acids of environmental relevance or concern. The PVA-coated capillary reduced sorption especially of phosphorous containing analytes, which are prone to interact with the capillary surface similar to glyphosate. For most analytes, the LODs in aqueous media and beer matrix were in the lower μg/L range, except for phosphonic acid and oxamic acid, which suffer signal intensities by adduct formation with sodium formate. In case of TFA, tremendous background noise deteriorated the LOD, which was not the case for DFA which has a roughly 20 times lower LOD. Severe matrix effects of analytes with low electrophoretic mobilities were observed; diluting or extracting the matrix via SPE can be an option to enhance signal intensities and thereby LODs; however, good recovery rates have to be ensured. The separation of the strong acids DFA and TFA was not sufficient. Just recently, Höcker et al. [103] demonstrated the separation of strong acids (e.g., chlorinated and brominated acetic acids) using a new CE-nanoESI-MS method with LOQs below 0.5 μg/L. For a broad screening of strong organic acids, further BGE modifications may be necessary, adapting pH and possibly the addition of organic solvents.

## Conclusion

A rapid, derivatization-free CE-MS method for GLP analysis in complex matrices was developed for different applications. The use of a PVA-coated capillary enabled selective GLP analysis with reduced sorption to the capillary wall and high separation efficiency. The use of volatile BGE components (formic acid and ammonia) enabled sensitive MS detection with LODs in the low μg/L range for GLP and AMPA. For GLP, the combination of an acidic BGE and negative ESI polarity provided high selectivity and high matrix tolerance. Divalent metal cations did not disturb the analysis, which is in contrast to LC methods with GLP derivatization. The combination of large volume injection with sample-induced ITP (natural phosphate in beer as transient leader) provided a simple and rapid online preconcentration method taking advantage from the high salt loads of several sample types. LODs below 2 μg/L and an accuracy of 94.3–110.7% in beer were reached. Beer samples were analyzed directly after degassing. No further sample preparation was necessary for precise GLP quantification using isotopically labeled standards. AMPA analysis is possible with this method with some restrictions as the compound was only transported by EOF within the sample plug. We demonstrated that the method is also applicable to other strong acids relevant in environmental samples such as TFA, DFA, MCPA, GLU, 3-MPPA, IDA, phosphonic acid, oxamic acid, and derivatives of GLP and AMPA. Hence, this method initially developed for GLP screening is also applicable for strong organic acids, especially in samples with moderate phosphate loads. Overall, the detection limits reached are well suited to match the maximum contaminant limits of GLP in water in the USA and partly for foodstuff in the EU. Future investigations will address GLP and AMPA extraction and quantification in different food and environmental samples.

## Electronic supplementary material

ESM 1 (PDF 502 kb).
