# Supplementary material for: Capillary electrophoresis-mass spectrometry for the direct analysis of glyphosate: method development and application to beer beverages and environmental studies
Source: Anal Bioanal Chem. 2020 Jun 10;412(20):4967–83. doi: 10.1007/s00216-020-02751-0 (PMC7334262; doi:10.1007/s00216-020-02751-0)
Supplement: Supplementary file 1 — (PDF 502 kb). [file 216_2020_2751_MOESM1_ESM.pdf]

Analytical and Bioanalytical Chemistry

Electronic Supplementary Material

Capillary electrophoresis-mass spectrometry for the direct analysis of glyphosate: method development and application to beer beverages and environmental studies

Benedikt Wimmer, Martin Pattky, Leyla Gulu Zada, Martin Meixner, Stefan B. Haderlein, Hans-Peter Zimmermann, Carolin Huhn

Additional figures for the method development for glyphosate analysis by CE-MS in different matrices, showing: S1) open tubular chromatography on an OHNOON-coated capillary with discussion; S2) optimization of the tip geometry of the platinum-iridium ESI needle for enhanced electrospray stability; S3) glyphosate signal parameters for different injection volumes; S4) isotachophoresis of counter ions from sheath liquid into the capillary, induced by different sheath liquid conditions, and S5) large volume injection of an organic beer sample spiked with glyphosate close to the method's LOD. Additionally, matrix effects of beer sample are summarized in Table S1.

A) Coatings

Cationic coating: To avoid glyphosate sorption via the phosphonate group and thus via hydrogen bonds, the first coating strategy investigated was to provide a capillary surface modification having fewer sites for hydrogen bonding, evoking a high negative EOF using the cationic OHNOON coating [Pattky, M.; Huhn, C. *Anal Bioanal Chem* **2013**, *405*, 225-237]. Ionic interaction was reduced using BGEs at very low pH (here pH 2.8), where glyphosate has only little negative charge (charge number approximately –0.52/–0.44). However, a very pronounced interaction between the OHNOON coated surface and glyphosate, AMPA and glufosinate occurred, evidenced by the absence of any glyphosate signal even after 20 min electrophoresis at −30 kV, despite coelectroosmotic migration. Upon flushing an aqueous plug of glyphosate, AMPA, and glufosinate through the capillary with 1 bar inlet pressure without voltage application, all compounds were detected separated, though especially glyphosate with inacceptable peak shape: An open tubular chromatography was present demonstrating the strong interaction of all compounds with the coating material.

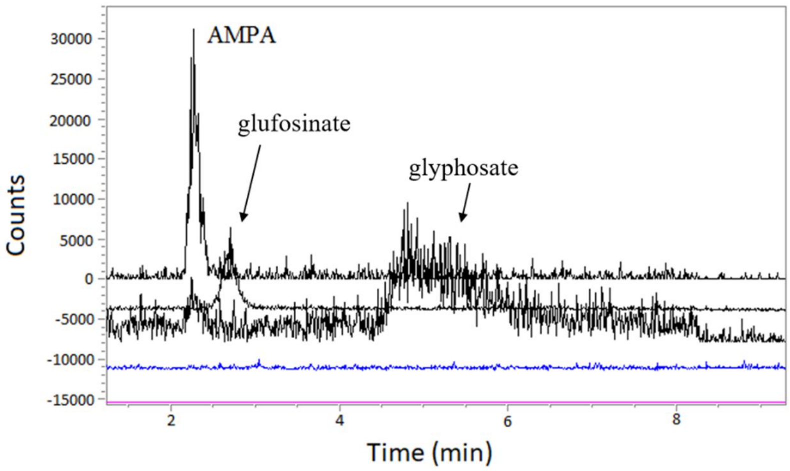

Fig. S1 Open tubular chromatogram of glyphosate, glufosinate and AMPA on an OHNOON-coated capillary. The analyte mixture was injected at 75 mbar for 10 s. The capillary was then flushed with running buffer at 1 bar pressure without voltage application. The running buffer was 0.75 mol/L acetic acid with 0.25 mol/L formic acid

B) Platinum-iridium ESI needle

Different strategies to improve the tip of the electrospray needles were tested. Figure S2 shows the ESI tip as obtained from the manufacturer. The thickness of the needle reduces the sheath gas flow and impairs signal intensities. In addition, changes in migration times compared to measurements with a standard ESI steel needle were observed. Sharpening the needle tip by mechanical grinding improved the processes, however, mechanical grinding and polishing helped to obtain satisfactory results.

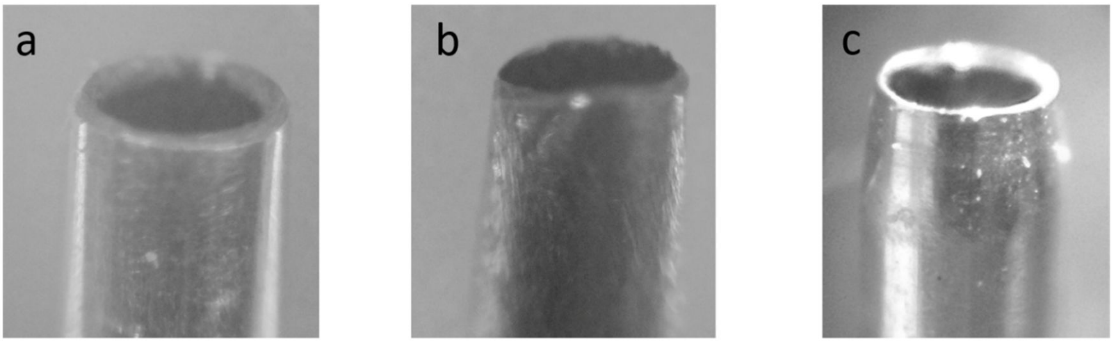

Fig. S2 Tip of differently prepared platinum-iridium-needles. (A) unmodified (as provided by manufacturer); (B) sharpened by mechanical grinding, and (C) mechanically grinded and polished (by goldsmith Ulrich Wehpke, Krefeld)

C) Optimization of pH and ionic strength of the BGE

The BGE was optimized with regard to pH (2–10) and ionic strength (formic acid (FA) concentration 33–250 mmol/L, titrated with ammonia to the desired pH). Exemplary electropherograms are shown in Figure S3. For discussion see figure legend and main text.

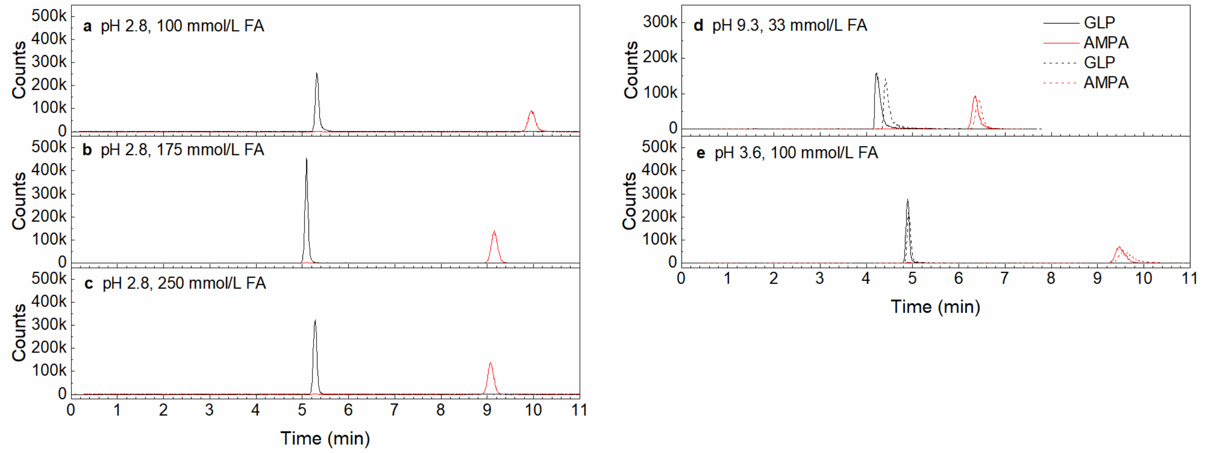

Fig. S3 EICs of glyphosate (GLP) and AMPA (each 10 µM, 1.69 and 1.11 mg/L, respectively) separated on a PVA-coated capillary (length 65 cm, i.d. 50 µm) using BGEs of ammonium formate (FA titrated with aqueous ammonia) with different pH and ionic strength (see figure legend). The BGE was used for 10 runs, representative EICs are shown. For pH 9.3, only 7 runs were executed successfully, with the last run shown in dashed lines. For pH 3.6, signal broadening of AMPA increased with run number, the 5th run is shown in dashed lines. Injection was accomplished at 50 mbar for 5 s, separation voltage was –30 kV with 50 mbar pressure. Sheath liquid was isopropanol:water 1:1 with additional 0.1 % FA, flow rate 0.5 mL/min (1:100 split)

D) Acidic vs. alkaline sheath liquid conditions

To understand effects induced by the counter ion present in the sheath liquid (SL) (proton in case of formic acid (FA), or ammonium in case of ammonia (NH_3)), we added different concentrations of FA or ammonia to the SL (isopropanol:water ratio 1:1), and observed conductivity changes within the capillary using a C^{4}D-detector at an effective length of ca. 55 cm. In case of a BGE consisting of 175 mM and 40 mM NH_3 (regardless of the presence of ammonia in the SL), the conductivity signal (Figure S4, solid lines) shows a steep signal decrease at approx. 4 min due to an ITP system migrating from the MS towards the CE-inlet. As visible from Figure S4, when both BGE and SL were void of ammonium ions (dashed black line in Figure S4), a “normal” electropherogram is observed, only showing the passage of the sample plug through the detection window (at minute 12) by the action of the EOF and suction effects.

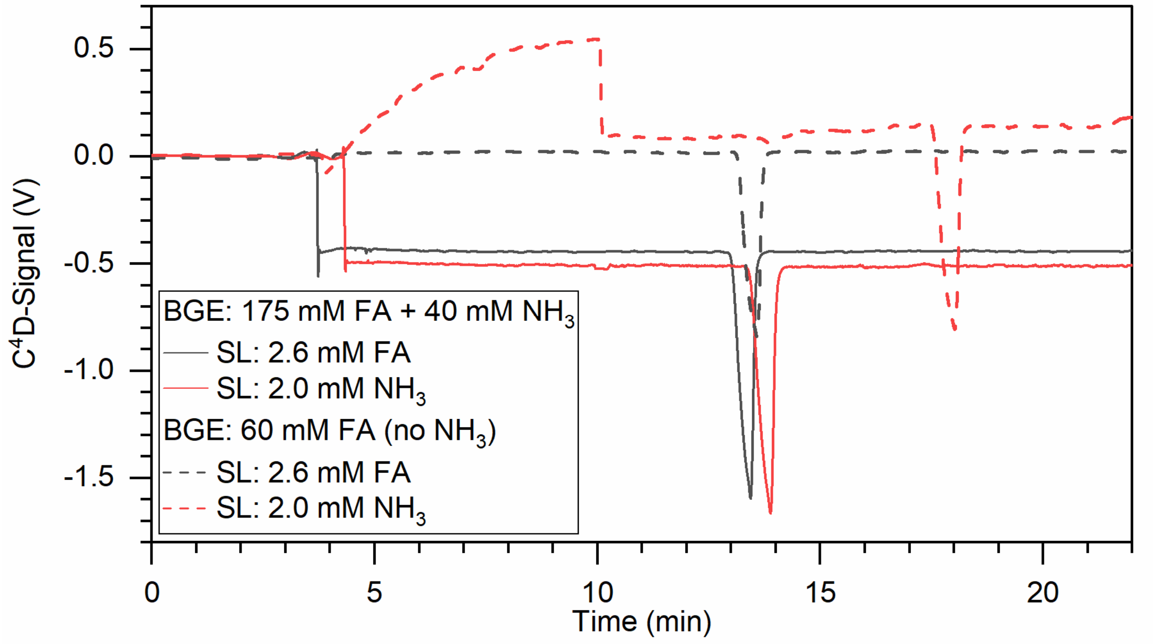

Fig. S4 Contactless capacitively coupled conductivity detector (C^4D) (Edaq, Australia) signal at an effective length of approximately 55 cm separation length (68 cm total capillary length). Solid lines: BGE made of 175 mM FA and 40 mM ammonia (pH 2.8) with either an acidic SL (addition of formic acid, black lines) or a basic SL (addition of ammonia, red lines). Dashed lines: the BGE is made of 60 mM FA without ammonia (pH 2.3)

We explain the experimental findings as such: For a system with ammonium ions present in the BGE but not the sheath liquid (solid black line), a sharp ITP boundary with NH_4^+ of the BGE as leading ion and protons from the SL as terminating ions. This boundary migrates from the MS towards capillary inlet (counter ITP, see [Melzer et. al, *Electrophoresis*, **2020**; DOI: 10.1002/elps.201900454]). The movement of the counter ITP changes local pH and conductivity and thus, of course also analyte migration upon passage. It is important to note, that this effect is also present, when ammonia is added to the SL (solid red line) as concentration differences and mobility differences are present for ammonium in the BGE and in the SL, so that an ITP system with a reduced mobility window is formed [Malá et al. *Electrophoresis*, **2013**; *34*, 777-84].

When the BGE is void of ammonia but not the SL (red dashed line), ammonium ions will enter the capillary and migrate towards the inlet, replacing protons. The conductivity increases due to ammonium ions, the increase in the local pH leading to dissociation of FA (see incrase in detector signal in Figure S4 (dashed red line)). This is a dynamic process, visible in the current profile of the CE separation, which shows a steady increase during the first minutes of the separation. The drop observed at about 10 min in Figure S4 is difficult to explain withour further work. It may stem from changes in the degree of formic acid, changes in the velocity of the counter ITP or is due to instable/changing electrospray conditions.

E) Preconcentration via large volume injection and sample-induced transient isotachophoresis

Figure S5 shows some electropherograms from the analysis of Fidelio organic beer spiked with glyphosate. The injection pressure was 100 mbar, the injection time was varied between 30 and 50 s. For further discussion, see main text.

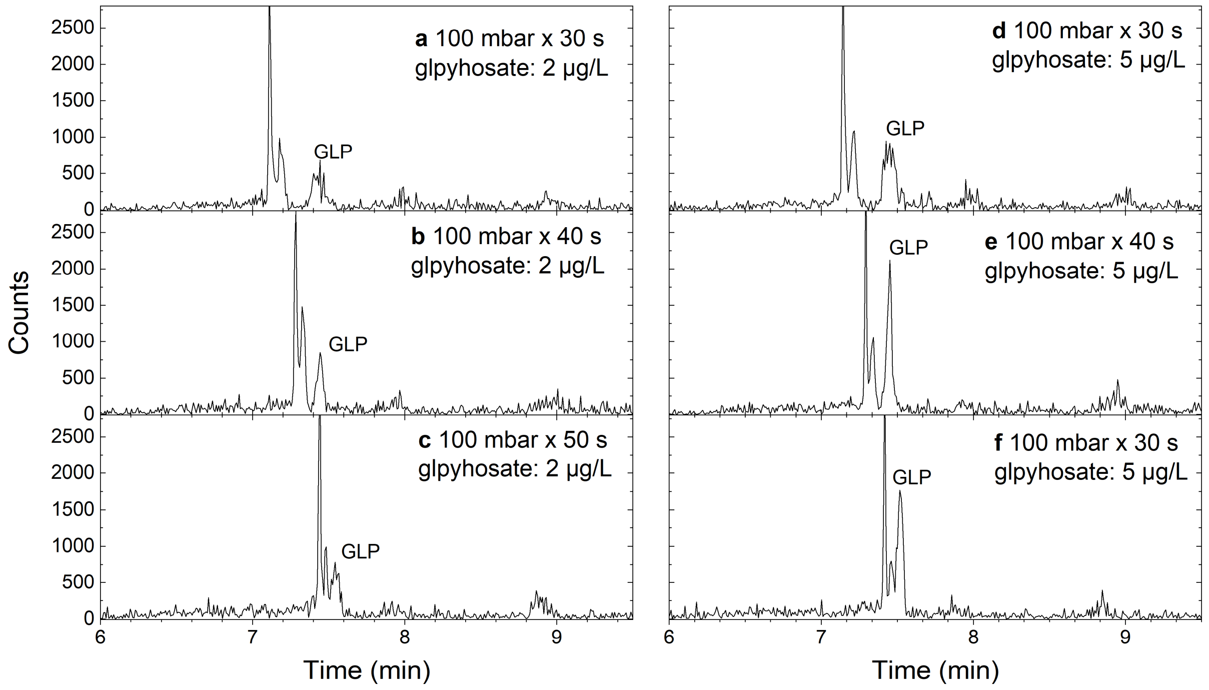

Fig. S5 Large volume injection of Fidelio organic beer, (a) to (c) spiked with 2 µg/L and (d) to (f) with 5 µg/L glyphosate (GLP). LVI was accomplished with 100 mbar for 30 s (a and d), 40 s (b and e) and 50 s (c and f). Separation was achieved on a PVA coated capillary (i.d. 50 µm, length 65 cm) using a BGE with 175 mM formic acid titrated to pH 2.8 with ammonia, and a separation voltage of –30 kV with additional 30 mbar pressure. Within the first two minutes of the separation, the voltage was ramped from –15 kV to –30 kV. Sheath liquid was isopropanol:water 1:1 with additional 0.01 % formic acid

F) Signal area repeatability for glyphosate and AMPA in water and beer plus matrix effects at different concentrations

Table S1 Relative standard deviation of signal area (RSD Area) for selected concentrations used for calibration (5, 10, 30, 50, 70, 100, 200, 500, and 700 µg/L; *n* = 3) of glyphosate and AMPA in aqueous solution and beer matrix. The matrix effect for glyphosate in beer samples was calculated for each analyte concentration by (average signal area matrix)/(average signal area aqueous solution) x 100 %

	glyphosate			AMPA		
	water	beer matrix		water	beer matrix	
	RSD Area (%)	RSD Area (%)	matrix effect (%)	RSD Area (%)	RSD Area (%)	matrix effect (%)
5	24.2			56.1		
10	10.6	9.1	119.0	2.6		
30	11.2	7.0	113.3	3.4	8.8	9.4
50	5.2	3.0	116.8	9.0	4.8	10.8
70	9.2	2.1	119.8	2.4	14.1	13.4
100	4.8	1.1	107.4	7.9	8.8	12.0
200	1.0	0.8	106.7	6.6	6.1	12.2
500	1.8	1.4	99.5	1.8	0.8	10.1
700	4.3	1.4	77.4	2.7	3.8	9.1
